# Evolutionary perspectives, heterogeneity and ovarian cancer: a complicated tale from past to present

**DOI:** 10.1186/s13048-022-01004-1

**Published:** 2022-06-03

**Authors:** Patriciu Achimas-Cadariu, Paul Kubelac, Alexandru Irimie, Ioana Berindan-Neagoe, Frank Rühli

**Affiliations:** 1 Department of Surgery, The Oncology Institute ‘Prof. Dr. Ion Chiricuta’, 34-36 Republicii street, 400015 Cluj-Napoca, Romania; 2 Department of Oncology, Iuliu Hatieganu University of Medicine and Pharmacy, Cluj-Napoca, Romania; 3 Department of Medical Oncology, The Oncology Institute ‘Prof. Dr. Ion Chiricuta’, Cluj-Napoca, Romania; 4 Research Centre for Functional Genomics, Biomedicine and Translational Medicine, Iuliu Hatieganu University of Medicine and Pharmacy, Cluj-Napoca, Romania; 5 Research Center for Advanced Medicine Medfuture, Iuliu Hatieganu University of Medicine and Pharmacy, Cluj-Napoca, Romania; 6 Department of Functional Genomics and Experimental Pathology, The Oncology Institute ‘Prof. Dr. Ion Chiricuta’, Cluj-Napoca, Romania; 7 Institute of Evolutionary Medicine, Zurich, Switzerland

**Keywords:** Ovarian cancer, Clonal evolution, Spatial heterogeneity, Temporal heterogeneity, Survival

## Abstract

Ovarian cancer is composed of a complex system of cells best described by features such as clonal evolution, spatial and temporal genetic heterogeneity, and development of drug resistance, thus making it the most lethal gynecologic cancer. Seminal work on cancer as an evolutionary process has a long history; however, recent cost-effective large-scale molecular profiling has started to provide novel insights coupled with the development of mathematical algorithms. In the current review, we have systematically searched for articles that focused on the clonal evolution of ovarian cancer to offer the whole landscape of research that has been done and highlight future research avenues given its characteristic features and connections to evolutionary biology.

## Introduction

Worldwide, each year more than 300.000 new cases of ovarian cancer are diagnosed and 185.000 patients succumb to their disease [1], without any major improvement in the long-term overall survival over the past three decades, despite improved disease control rates measured as 5-year overall survival [2].

As Theodosius Dobzhansky said in a seminal paper in 1973 that “Nothing in biology makes sense except in the light of evolution” [3], Darwinian principles applied in cancer science have brought much to our current understanding of this disease, and ovarian cancer makes no exception [4, 5]. The high incidence of ovarian cancer can also be attributed to an evolutionary mismatch to our rapid social evolution. The rising incidence in industrialized societies can be partly explained by reproductive patterns such as increased total number of ovulations, increased age at first birth, fewer pregnancies [6, 7], and a prolonged estrogen exposure [8] with partial attenuation through the introduction of oral contraceptives but predicted increases for the following years [9]. Interestingly, the high prevalence of founder BRCA1/2 mutation carriers can be explained by their increased lifetime reproductive success in natural fertility conditions that also masked their detrimental oncogenic potential for cancers of the reproductive tract [10, 11].

Within its natural history, ovarian cancer is generally a disease that remains localized to the peritoneal cavity throughout its course, with occasional distant metastases. With vague and nonspecific signs and symptoms, the initial diagnosis is usually delayed until the occurrence of extensive intra-abdominal spread through the contiguous peritoneal surfaces, ascites fluid, and rich lymphatics. Death usually occurs through progressive inanition and gastrointestinal tract obstruction that cannot be corrected through surgery due to extensive carcinomatosis [7].

Ovarian cancer should be regarded as not one but many diseases. Several histological subtypes have been described, with high-grade serous carcinoma as the most commonly diagnosed. However, its exact point of origin is still a matter of ongoing debate [12], and in-depth transcriptional analysis by The Cancer Genome Atlas project has defined four different transcriptional subtypes [13]. Still, the established standard strategy for treating advanced ovarian cancer has been maximum cytoreductive surgery and platinum based chemotherapy followed by surveillance for potential recurrence [14]. Complete debulking to no residual (0 mm vs 1–10 mm) was associated with improved overall survival and also impacted outcomes after the occurrence of relapsed disease, probably through the physical depleting of the reservoir of chemotherapy resistant clones. Neoadjuvant chemotherapy (NACT) followed by interval debulking surgery (IDS) is an option for treating patients with advanced bulky disease where upfront primary debulking surgery (PDS) is not technically feasible [15]. There is still doubt if the survival advantage of complete debulking is the same whether through PDS or IDS. Two randomized trials have shown similar survival rates for PDS and IDS, but recent evidence suggests that IDS correlates with a higher risk of developing platinum resistance [16]. This is most likely explained through the exposure of a high tumor volume with multiple tumor subclones to the stringent selection pressure of chemotherapy with subsequent expansion of resistant clones [17, 18]. The incorporation of antiangiogenic agents to standard therapy has brought only minor increments in PFS, while the addition of PARP inhibitors (PARPi) as maintenance therapy in BRCA mutated patients has significantly prolonged PFS with OS results still not mature [19, 20].

Despite high initial response rates, all too often relapse occurs, and subsequent treatment strategies maximize quality and length of life but are less likely to be curative. Rechallenge with platinum-based chemotherapy depends on the platinum free interval while surgery is limited to a subset of patients where OS results are still pending [21]. If not present from the first relapse, after several lines of treatment platinum resistant disease develops and represents a daunting clinical entity with limited therapeutic options and an overall survival of under 12 months [22]. Interestingly, about 15% of patients survive more than ten years however survivors of advanced stage disease represent a heterogeneous group that we have not yet determined or understood what makes them long-term survivors with more research needed for an understanding of this particular group [23].

Many of the clinical aspects previously presented depict evolutionary concepts such as spatial heterogeneity, temporal heterogeneity, and system induced selection pressure. Our current understanding of cancer has recently seen an exponential growth with the continuous technological development that offered the necessary tools to more precisely infer tumor cell dynamics. Hence, in the current review, we have systematically searched for articles that focused on the clonal evolution of ovarian cancer in an effort to offer the full landscape of research that has been done and highlight future research avenues given its characteristic features and connections to evolutionary biology. In the context that ‘Evolution has no eyes to the future’ [24] perfectly applies to the interaction between tumour and host microenvironment, we envision that using evolutionary principles we could be able to understand better the processes that drive tumor heterogeneity and select anticipative therapeutic strategies for improving patients’ outcomes.

## Methods

The present systematic review was written in accordance with the Preferred Reporting Items for Systematic Review and Meta-Analysis Protocols statement. This review was also registered at PROSPERO under registration number CRD42018105413.

A comprehensive search of English written articles was performed on Web of Science – Science Citation Index Expanded, PubMed, EMBASE with no date restriction until July 2018. Secondary references were identified through screening of the reference lists of relevant studies. The following headings were used in the search strategy, including closely related words: genetic heterogeneity, clonal evolution, biological evolution, ovarian cancer. The detailed search strategy is presented in Table 1. After retrieving all articles generated by the search strategy and excluding duplicates, titles and abstracts were evaluated for eligibility. Included studies were restricted to human tissue, pathologically confirmed as epithelial ovarian cancer, and had a minimum of two paired samples per case. Subsequently, full text articles were retrieved and assessed for eligibility using the same search criteria, detailed in Tables 2 and 3.

**Table 1:** Detailed search strategy

Database	Search syntax
Web of Science Science Citation Index Expanded (SCI-EXPANDED) –1975-present	(((TS = biological evolution) OR (TS = biologic^*^ evolut^*^)) OR ((TS = clonal evolution) OR (TS = clonal^*^ evolut^*^)) OR ((TS = Genetic Heterogeneity) OR (TS = Genetic^*^ Heterogen^*^))) AND (TS = ovarian cancer) AND LANGUAGE: (English) Indexes = SCI-EXPANDED Timespan = All years
Pubmed	(((((((((Genetic^*^ Heterogen^*^) OR biologic^*^ evolut^*^) OR clonal^*^ evolut^*^) OR genetic heterogeneity) OR biological evolution) OR clonal evolution)) AND ovarian cancer)) AND English[Language]
Embase	[embase]/lim NOT [medline]/lim AND (‘ovarian cancer’/exp OR ‘ovarian cancer’) AND (‘genetic^*^ heterogen^*^’ OR ‘biologic^*^ evolut^*^’ OR ‘clonal^*^ evolut^*^’ OR ‘genetic heterogeneity’/exp OR ‘genetic heterogeneity’ OR ‘biological evolution’/exp OR ‘biological evolution’ OR ‘clonal evolution’/exp OR ‘clonal evolution’) AND ([article]/lim OR [article in press]/lim) AND [english]/lim

**Table 2:** PRISMA flowchart

1. Identification	Records identified through database searching (*n* = 1663) WOS – SCIE: 735 Pubmed: 605 Embase: 323	Additional records identified through other sources (*n* = 29)
	Records after duplicates removed (*n* = 1523)	
2. Screening	Records screened (*n* = 1523)	Records excluded (*n* = 1432)
3. Eligibility	Full-text articles assessed for eligibility (*n* = 91)	Full-text articles excluded (*n* = 62)
4. Inclusion	Studies included in qualitative synthesis (*n* = 29)

**Table 3:** Summary of included articles in the systematic review

First author	Year	Cohort	Method	Conclusion	Ref
Taylor HC	1959	550 samples from 36 patients and 12 temporal paired samples	Light microscopy	Consistent morphology across tumor areas and at relapse	[25]
Knoerr-Gaertner H	1977	4 cases of bilateral cystadenocarcinomas	Chromosomal banding	Identical chromosomal markers found in bilateral cystadenocarcinomas without definite proof for clonal or multicentric evolution	[26]
Roberts CG	1990	3 samples from 1 case	Chromosomal banding and inferred clonal cytogenetic evolution	First report of clonal evolution based on chromosomal breakpoints analysis	[28]
Pejovic T	1991	34 samples from 15 cases	Chromosomal banding	No evolution seen in spatially separated samples. Possible explanations given: metastatic spread is a late event in evolution, identical evolution in paired samples, clonal dominance of cells with growth advantage	[27]
Vandamme B	1992	17 samples from 6 cases and 4 temporal samples from 1 case	Restriction fragment length polymorphism	11p deletion is a late event in disease progression	[30]
Abeln ECA	1994	3 cases	Flow cytometry and PCR on microsatellite markers	Proof of principle PCR-based LOH detection on flow sorted tumor cells can determine their monoclonal origin and can be used for the analysis of tumor heterogeneity	[32]
Deger RB	1997	21 samples from 8 cases	Chromosomal banding	50% of cases had less complex chromosomal aberrations in metastatic lesions	[34]
Ioakim-Liossi A	1999	9 patients with paired temporal samples	Chromosomal banding	Identical chromosomal aberrations after disease progression or treatment administration	[29]
Zborovskaya I	1999	18 samples from 9 cases	PCR on microsatellite markers	In 5 out of 9 cases the LOH patterns were different in the primary tumor and the metastatic nodes	[33]
Fishman A	2005	14 samples from 7 cases	Chromosomal comparative genomic hybridization	6 out of 7 cases presented less chromosomal aberrations or normal genomes in metastatic sites than in paired primary lesions	[35]
Khalique L	2007	110 samples from 16 cases	PCR on microsatellite markers, multiplex SNP analysis, maximum parsimony tree analysis	All cases are monoclonal in origin and display extensive intra-tumor heterogeneity	[41]
Khalique L	2009	186 samples from 22 cases	PCR on microsatellite markers, maximum parsimony tree analysis	Monoclonal origin but become polyclonal with different clones gaining metastatic potential during early and late stages of evolution. There is extensive heterogeneity between metastatic samples with no significant differences from primary tumor	[42]
Cooke SL	2010	3 platinum sensitive and 4 platinum resistant cell lines derived from 3 patients, 6 paired pre/post neoadjuvant chemotherapy samples	Array CGH, M-FISH, Array SNP	Platinum resistance develops from pre-existing minor clones but no enrichment is observed after short-term chemotherapy	[36]
Micci F	2010	70 samples from 32 cases	Chromosomal banding, HR-CGH	Metastatic spread to the contralateral ovary is a late event in evolution	[31]
Ng CK	2012	2 platinum sensitive and 2 platinum resistant cell lines from 2 patients	NGS	Tandem duplicator mutator phenotype is an ongoing phenotype that arose early before divergence of platinum sensible/resistant lineages and might represent a novel DNA repair defect independent of HR	[37]
Bashashati A	2013	29 samples from 5 cases and 2 paired temporal samples from 1 case	NGS, SNP array, RNA array	Extensive heterogeneity at all levels with early divergence and highly individual evolutionary trajectories. TP53 is the only consistent mutation across samples. Cell free DNA offers a narrow and heterogeneous picture of common ancestral mutations. Stable clonal population at relapse might be a feature of long survivorship	[43]
Castellarin M	2013	6 paired temporal samples from 2 cases	NGS	89% of mutations found in relapsed tumors are present in primary tumors. Predominant TP53 mutations in all samples	[38]
Tone AA	2014	19 primary and 18 recurrent samples from 11 cases	Cancer hotspot NGS	Almost half of patients with low grade SOC had RAS/RAF pathway mutations. Among them, 20% exhibit spatial and temporal heterogeneity	[48]
Lee JY	2015	11 samples from 1 case	NGS, SNP array	Early divergence of primary clusters, metastatic cluster accumulated few mutations. Only 6% shared somatic mutations across all samples except TP53	[44]
Mota A	2015	6 primary and 6 recurrent samples from 1 case	NGS, CGH	Significant mutational divergence, with 41% shared somatic variants between 1 primary and 2 recurrent samples	[39]
Schwarz RF	2015	135 samples from 14 cases	SNP array, NGS, Digital PCR, MEDICC algorithm	Potentially unknown mutator phenotypes lead to non-clock-like evolutionary trajectories. There are only minor genomic changes (in comparison with the overall changes) with neoadjuvant chemotherapy, with an average of 46 new genomic events. Patients with high clonal expansion have a shorter survival. Marked early divergent evolution of relapsed samples	[49]
Eckert MA	2016	32 samples from 8 patients	NGS	Various anatomic sites have similar mutation burden indicative of early establishment of genomic instability. STIC can represent primary or metastatic lesions	[45]
Lambrechts S	2016	31 cases with paired primary and recurrent samples	SNP array, NGS	Huge variability between primary and recurrent tumors with 58% shared mutations between primary and recurrent samples. TP53 was the most frequently observed (18 out of 23 pairs, 78%) with few other recurrent mutations	[40]
McPherson A	2016	68 samples from 7 cases	NGS, Single cell sequencing	Metastatic sites usually comprise oligoclonal populations with at least one polyclonal site in each patient. Two distinct modes of peritoneal spreading: monoclonal, unidirectional and polyclonal with reseeding	[50]
Choi YJ	2017	25 samples and ascitic cells from 4 cases	NGS, microarray CGH, methylation profiling	Ascitic cancer cells diverge early through a polyseeding mechanism and accurately reflect common somatic mutations, CNAs and DNA methylations in advanced ovarian cancer	[53]
Yin X	2017	22 bilateral samples from 11 cases	NGS	Bilateral ovarian cancer lesions have a common ancestor but diverge early and display significant heterogeneity	[46]
Arend RC	2018	Paired pre/post NACT samples from 19 patients, 14 paired plasma samples	NGS 50 genes, Gene expression panel	Targeted sequencing of cell free DNA was not informative for NACT tumor induced changes	[52]
Chien J	2018	9 samples for each of the 4 patients	RNA seq	Peritoneal metastases arise early and are composed of multiple subclones, with shared SNV at all regions of one patient 2.13–25%	[47]
Zhang AW	2018	212 samples from 38 cases	NGS, Gene expression, IHC	Within patient spatial immune variation influences the dissemination of malignant clonal populations	[51]

## Results

### Early inferences of tumor heterogeneity

More than six decades ago, clinicians were asking to some extent, the same clinical questions as we do today but to a greater depth regarding ovarian cancer: “Do the cells of the metastasis or the recurrence behave as did the primary? Does the apparent acceleration in the downhill course of the patient depend upon an increase in the intrinsic malignancy of the tumor?”. The authors analyzed a number 550 samples from different areas of 36 patients and 12 temporally paired cases were evaluated by the authors in light microscopy, concluding that in most cases, the tumor structure remained unchanged [25].

Cytogenetic studies demonstrated that chromosomal abnormalities precede histologic changes. There was evidence for the same stem lines with identical chromosomal changes in bilateral cystadenocarcinomas, but without the possibility of drawing a conclusion towards a common ancestor hypothesis or a parallel malignant process in both ovaries, although the authors favored the latter given the similar pattern seen in bilateral cystadenomas [26]. Another cytogenetic study on 34 samples from 15 patients identified identical karyotypes in primary and metastatic samples from the same patient, without any evidence towards an increase in cytogenetic diversity during tumor progression [27]. Through the technique of inferred clonal cytogenetic evolution, a study conducted on three spatially separated samples of ovarian carcinoma from the same patient demonstrated the clonal evolution in ovarian cancer by mapping the frequency of occurrence of 18 different chromosomal breakpoints [28]. Performing repetitive karyotyping of malignant effusions during disease progression or after treatment administration in 9 patients evidenced aneuploidy, karyotyping diversity, and double minute chromosomes but in paired samples reported there were identical chromosomal alterations [29].

The use of restriction fragment length polymorphism probing in 7 patients demonstrated the coexistence of malignant cell clones, and the deletion of chromosome sequence 11p13-11p15.5 was considered a late event in disease progression [30]. Similar results were subsequently obtained in a larger series and with the addition of high-resolution comparative genomic hybridization (CGH), showing that metastases to the contralateral ovary had occurred as a late event in the clonal evolution [31].

A proof of principle study using PCR-based loss of heterozygosity (LOH) detection on flow sorted tumor cells demonstrated the feasibility of this method to confirm the monoclonal origin of different tumor cell populations and may be helpful in reconstructing the clonal evolution in solid tumors [32]. The evaluation of 10 microsatellites through PCR on 9 cases with primary tumors and paired metastases found an identical LOH spectrum in 4 cases, while in 5 cases the LOH patterns were different in the primary tumor and the metastatic nodes [33]. A study conducted on 8 cases with 21 samples showed that in 4 cases, the number of chromosomal aberrations in the metastatic site was lower than in the corresponding primary tumor site, in contradiction with the expected evolutionary finding [34]. Fishman et al. used comparative genomic hybridization to analyze the chromosomal profile of seven primary high grade serous ovarian cancer tumors and their paired metastases. A wide range of genetic alterations were present in the primary tumors however in 6 out of 7 metastatic lesions there were fewer genetic alterations or normal genomes, suggestive in the author’s opinion that this might reflect not ordinary metastases migrated from the primary tumor but developed independently as de novo carcinogenesis [35].

### Molecular inferences of temporal heterogeneity

One of the first studies that analyzed in three cell line series the genetic changes associated with the transition from platinum sensitive to platinum resistant disease suggested they were not linearly related, and that platinum resistant disease emerges through the outgrowth of a pre-existing platinum resistant subclone under the selective pressure of treatment. Vast differences between sensitive and resistant clones were confirmed through multicolor fluorescence in situ hybridization and array CGH, with a higher genomic complexity at presentation than at relapse. A similar analysis of 6 paired tissue samples taken before and after three cycles of neoadjuvant chemotherapy revealed very few differences. The lack of differences after neoadjuvant chemotherapy could be attributed to a short exposure to treatment, survival of sensitive clones due to environmental reasons, or to the presence of a dominant clone at presentation [36]. Next generation sequencing of two of the above samples identified besides loss of homologous recombination (HR), that the tandem duplicator mutator phenotype is an ongoing mutator phenotype that arose early before lineage divergence. Its persistence may be responsible for the continuous evolution and might represent a novel, unknown deficit in DNA repair different from HR, with an estimated frequency of 12.8% [37]. Performing whole exome sequencing on ascites derived tumor cells at three time points found that besides TP53 mutations that were present at all time points, 89% of mutations found in recurrent tumors were also present at the beginning. This is concordant with previous reports that recurrent disease arises from the selective pressure of chemotherapy on pre-existent clones, even after two lines of chemotherapy [38]. A similar report underscored the situation in which the primary tumor is composed of mutationally heterogeneous clones, some of which give rise to the recurrences, with 41% shared somatic variants between 1 primary and 2 recurrent samples [39]. An extensive study that analyzed 31 paired primary and recurrent samples found extreme variability in heterogeneity within tumor pairs, likely caused by branched evolution in the primary tumor of a platinum resistant subclone that causes subsequent relapse. An average of 47 non-synonymous confirmed somatic mutations per tumor pair (range 5–147) were observed, with TP53 as the most frequently observed in 78% of cases, but few other genes were recurrently mutated. Out of the 1074 mutations, 58% were shared, whereas 15% (range 0–42%) and 27% (range 0–100%) were unique for the primary or recurrent samples. Similarly, 41% of the genome was affected in both primary and recurrent samples by copy number alterations. None of the clinical variables correlated with tumor heterogeneity. Interestingly, platinum sensitive tumors maintained HR deficiency when converting to a platinum resistant phenotype, suggesting that PARPi could be useful in this clinical situation, although they are currently approved only for platinum sensitive disease [40].

### Molecular inferences of spatial heterogeneity

One of the first studies that conducted a comprehensive evaluation of intra-tumor heterogeneity included 110 samples from 16 patients with advanced high grade serous ovarian cancer. Screening for genetic alterations was done using microsatellite analysis and single nucleotide polymorphism (SNP) analysis, with maximum parsimony tree analysis used to infer the clonal relationships. Both approaches reached the same conclusions that there is extensive intratumor heterogeneity between all regions of the same patient despite their similar morphological appearance. By reconstructing their evolutionary history a monoclonal origin was suggested with no evidence of two or more ancestral lines. Common alterations included deletions on chromosomes 13 and 17, where BRCA1/2 and p53 genes are also located [41]. Employing similar methods, a subsequent study was conducted by the same group and focused on the relationship between primary and metastatic lesions. The authors found no cases in which the genetic profiles of all the metastases of a patient were the same, and there were no significant differences in the level of genetic heterogeneity between metastatic samples and primary tumors. The data presented support a model with a common clonal origin that becomes polyclonal from which clones with different genetic backgrounds have the potential to metastasize during the early and late stages of genetic divergence [42].

An in-depth approach that evaluated the genomic diversity at nucleotide, copy number, and gene expression scales in 31 samples from 6 patients revealed individualized extensive intratumor heterogeneity. A range of 31–137 unique mutations/case was present with 51.5% (range, 10.2–91.4%) mutations present in all samples of a case. Except case 1, all other harbored a p53 mutation present in all samples, making it the most stable genomic feature. In one case, the fallopian tube lesion was a metastatic implant, whereas in another case, it harbored two dominant clones that gave rise to two histologically distinct populations that had a common ancestor, indicating the early occurrence of polyclonal subpopulations, thus complicating even more the evolutionary origin of ovarian cancer in the fallopian tubes. Two paired temporal samples with almost identical genomic mutations characterized a case with extended survivorship. Analysis of plasma cell free circulating tumor DNA detected a range of 1–12 mutations from the ancestral clone, illustrating a rather narrow and heterogeneous phenomenon of tumor DNA shedding across cases [43]. A study that analyzed a higher number of 11 spatially separated samples from an advanced stage high grade serous ovarian cancer reported a lower rate of 6% for shared somatic mutations in all samples, and there was an early divergence of two primary clusters with one of them leading to the formation of a metastatic cluster with little accumulation of somatic mutations [44].

Serous tubal intraepithelial carcinomas (STIC) possess most of the genomic aberrations of other intraperitoneal metastases and only in 4 out of 8 cases they represent the evolutionary precursor lesions, while other STIC lesions might actually represent metastases of other anatomic sites with patients specific mutational signature characterizing high grade serous ovarian cancer (HG-SOC) as a heterogeneous disease without a specific mutational signature except patient specific ubiquitous TP53 mutations [45]. Phylogenetic analysis of bilateral ovarian cancer samples demonstrated a common ancestry, and early disemination, with marked intra- and inter-tumor heterogeneity, as previously presented [46]. Another study that reconstructed the evolutionary history from the RNA of 4 patients from 9 spatially separated samples for each case reached similar conclusions with early branching of peritoneal metastases, and the presence of multiple subclones at each tumor implant [47].

Tumor heterogeneity has been less frequently described in low grade SOC, however, on a study on 11 cases, 1 in 5 (20%) patients with RAS/RAF pathway mutations exhibited spatial and temporal heterogeneity, despite not receiving targeted treatment against the mutation [48].

An in depth study using the MEDICC phylogenetic algorithm demonstrated that high intra-tumour heterogeneity measured through a clonal expansion index was associated with longer survival, supporting the hypotheses that clonal expansion is a surrogate for genetic diversity that favors the development of treatment resistant clones. Evolutionary clades in the patient specific trees often agreed with the anatomical sites where the sample was taken, supporting the physical shedding from the invasive lesions in the fallopian tube. In 8 out of 9 evaluable cases, cells retained their metastatic potential, and a model of metastasis to metastasis spread was supported with significant branching of tree topologies. Investigating whether evolutionary change occurs at a constant rate, the study found that 2 out of 14 patients had significant non-clock-like evolutionary trajectories with potentially unknown mutator phenotypes. Neoadjuvant therapy induced only minor genomic changes compared to the overall changes, with an average of 46 new events. Phylogenetic reconstruction of relapsed samples in 2 cases demonstrated their early divergence from the common ancestor. In one case NF1 deletion, while present in the dominant population at relapse, was already present at diagnosis in a minor proportion with subsequent clonal expansion [49].

A study that performed clonal population profiling of spatially distinct intraperitoneal clones (68 tumor samples from 7 patients) through whole-genome and single-nucleus sequencing identified evolutionary features such as mutation loss, convergent evolution and time dependent mutational signatures. Interestingly, metastatic sites were composed of clonally pure or highly related clones with at least one tumor site in each patient containing multiple subclones. In 5 cases, intraperitoneal spread was monoclonal and unidirectional, while two cases exhibited polyclonal spread and reseeding underscoring two different migratory patterns [50]. The same group of authors recently showed that among the reasons for non-random distribution of malignant clones into the peritoneal cavity are the immune related cells of the tissue microenvironment that seem to have a role in shaping the evolutionary history of cancer cells. The authors defined three patterns of tumor infiltrating lymphocytes (TILs), reflecting their density and distribution within the tumor microenvironment, with ES-TIL being the most immunogenic population (substantial epithelial and stromal TILs) in comparison with S-TIL (stromal TILs) and N-TIL (sparse TILs). Within the same patients extensive spatial variation was observed, with 17 out of 31 patients harboring more than one pattern of TILs. Using four different measures for assessing sample clone complexity it was evident that samples with ES-TIL elicit immune editing of subclonal populations through T Cell tumor clone tracking with subsequent expansion of tumor cell populations that harbor neoantigen loss and/or human leukocyte antigen LOH. However, multi-site TIL diversity also implies that immune deficient sites might represent cradles of clonal diversity for subsequent disease relapse. Another important aspect is that specific classes of genomic aberrations such as fold-back inversions that are present in a significant proportion of cases lead to poor immunogenic responses whereas homologous recombination deficient tumors are associated with upregulated imune pathways. Overall, patient specific spatial diversity of the tumor microenvironment significantly influences the intraperitoneal dissemination, offering a new perspective on HG-SOC clonal evolution [51].

The utility of using cell free DNA to monitor treatment induced genomic changes was assesed on 20 patients with paired pre/post NACT tumor and plasma samples through targeted next generation sequencing (NGS) and found that it was minimal and larger studies are needed to determine the role of cell free DNA in the management of HGSOC [52]. Given that multiregion sampling is not always feasible, a study on 4 patients evaluated if the genomic information extracted from ascitic cells can accurately reflect the tumor burden. The ascitic cells genomes included 84–100% of the common mutations and a considerable fraction (22.9–75.8%) of shared mutations that were present in at least two distinct samples, thus offering a large view of the mutational lanscape of advanced ovarian cancer. Inferring the phylogenies of ascitic cells in relation with spatially separated tissue samples demonstrated an early evolutionary divergence and polyseeding [53].

## Conclusions

Therapeutic strategies should be based on accurate knowledge of a tumor’s trajectory. It is obvious from the first published report that there were many questions regarding the heterogeneous clinical course of ovarian cancer however the lack of accurate tools to infer on its evolutionary history could not be surmounted even by a large number of evaluated samples, and no conclusions could be drawn except that in light microscopy in most cases there were no changes in tumor morphology [25].

In the following three decades, chromosomal banding techniques used in the study of spatially separated samples increased the analysis resolution. Similar complex chromosomal changes were observed in tumor samples, and there were no firm conclusions towards clonal heterogeneity [26]. It was suggested this was the result of a late metastatic process without any evolution after the emergence of the metastatic subclone, but the alternate hypotheses of an identical clonal evolution in both the primary and the metastatic lesions could not be excluded. Another proposed concept as a possible explanation for the identical chromosomal lesions seen in bilateral carcinomas was that of clonal dominance, the overgrowth of the primary tumor by cells that have a growth advantage [27]. In a proof of principle study, a diagram of the inferred cytogenetic changes of three spatially separated samples created a branching pattern for the clonal evolution of ovarian cancer [28]. This was in accordance with the general hypothetical model of clonal evolution presented by Nowel [54] and represented a new method that could be applied in the study of similar tumors from different patients or from sequential samples. Due to lack of genetic resolution, a study performing Giemsa banding chromosomal analysis of treatment or progression induced chromosomal changes reported the same clonal chromosomal aberrations [29].

Further studies that used more accurate techniques such as restriction fragment length polymorphism probing or high-resolution CGH identified the coexistence of malignant cell clones however the development of metastasis was considered a late event in evolution [30, 31]. After the introduction of PCR based LOH in ovarian cancer [32], a study based on a larger number of cases discovered a different spectrum of genetic alterations in metastases and confirmed the dissemination of only certain subclones [33], thus offering more precise interpretations of tumor evolution than previously studied based on chromosomal information [34, 35].

The advent of high throughput technologies demonstrated the existence of a common ancestor and revealed the scale of intratumor heterogeneity [41]. Analyzing the relationships between different metastatic samples of the same patient, there were no cases in which all metastatic samples of a patient were identical. It also became evident from the emerging data that it was in support of a model of clonal origin that soon after becomes polyclonal with different clones acquiring metastatic potential during early and late stages of genetic divergence [42–45, 47].

Extensive analysis of paired samples from diagnosis and recurrent disease showed that platinum resistant disease emerges from a minor pre-existent population through the selection pressure of chemotherapy with huge variability between the primary and recurrent disease [36, 38–40, 43, 46, 47], but a short administration of neoadjuvant chemotherapy didn`t seem to inflict significant genomic damage [36, 49]. Analysis of cell free DNA has been already tested in following the clonal dynamics of colorectal cancer patients [55]. Cells in the ascites fluid have been proven to reflect most the common somatic mutations of a patient as a potential future surrogate for monitoring the genomic burden of disease while circulating cell free tumor DNA has prooved non informative so far, owing to its small amount and presence of diluting nonneoplastic DNA [52, 53].

Subsequent analysis also showed that the presence of a tandem duplicator phenotype besides the well known homologous recombination deficiency as mechanisms that drive mutagenesis in a significant proportion of patients [37], suggesting that except TP53 other known actionable driver mutations are still elusive [38, 43, 44], contrary to the distinct entity of low grade serous ovarian cancer where cases with somatic mutations generally show stability across samples and time [48].

Previous observations that a stable genomic structure is associated with a longer overall survival [43] were confirmed through the phylogenetic quantification of heterogeneity that significantly predicted overall survival based on a clonal expansion index, in support of the hypotheses that high genetic diversity favors the development of treatment resistant disease [49].

Recent research has highlighted that most intraperitoneal mixtures are comprised in general of an oligoclonal population and at least one polyclonal site exists in every patient. Also, two non-random trajectories have been described, the first monoclonal and unidirectional and the second polyclonal with reseeding [50]. Theese patterns of spread seem to result from the spatial heterogeneity of the immune microenvironment that can actively shape the evolutionary history of cancer cells, with other clinical relevant interactions between mutator phenotypes and immune responses [51].

**Fig. 1:**
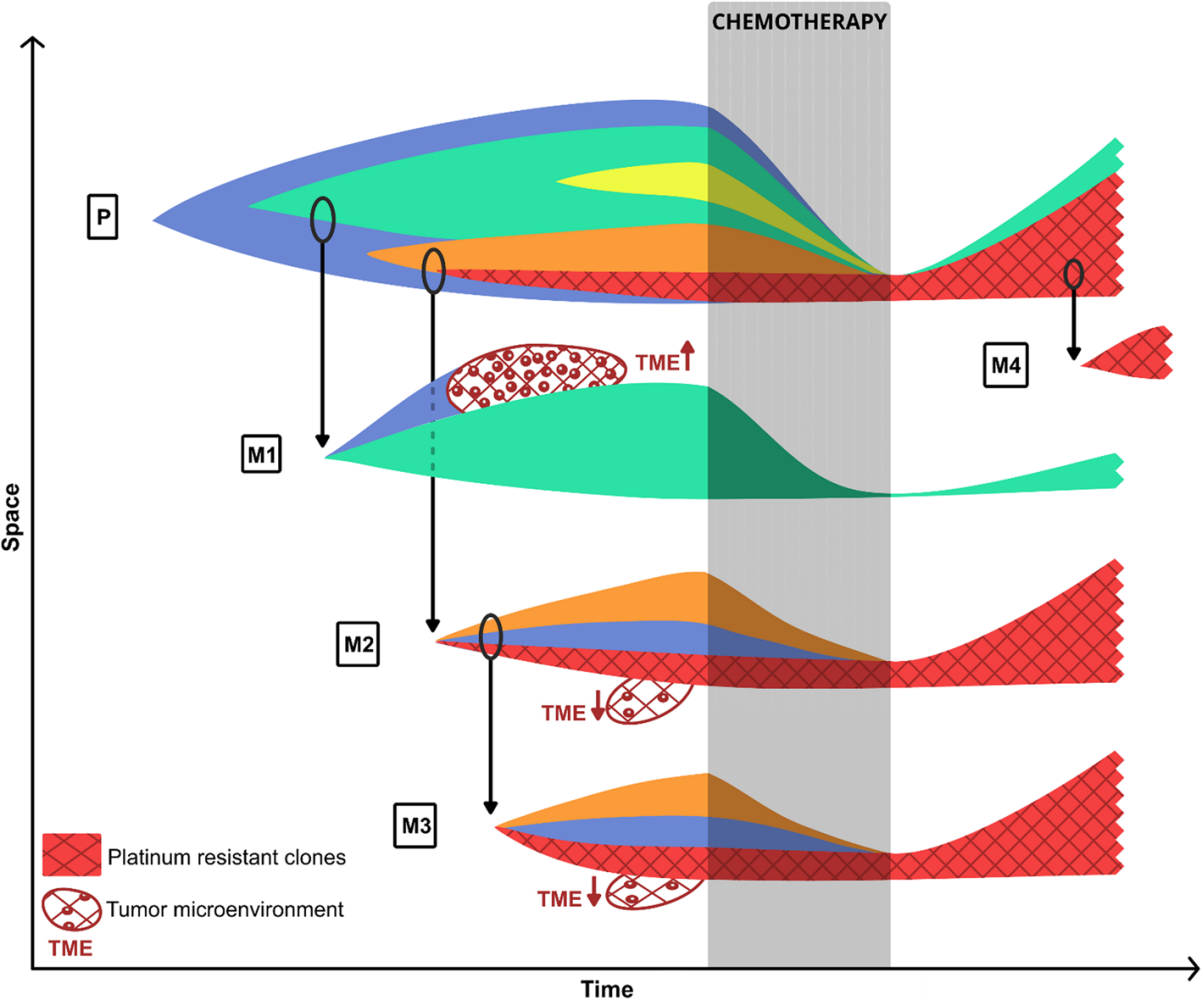
Concept of clonal progression in cancer. Primary ancestral clone (P) has divergent evolution with early (M1) and late (M2, M4) acquisition of metastatic potential and re-seeding of metastases (M3). A high immune infiltrated microenvironment shapes clonal evolution. Pre-existent platinum resistant clones drive tumor relapse

Cancer heterogeneity and cancer evolution represent a major challenge in front of effective therapy. A model of clonal evolution in ovarian cancer based upon some of the most important issues presented in this article is depicted in Fig. 1. Many of the published research on heterogeneity in ovarian cancer has been reffering to the genetic component, however heterogeneity in cancer is a more broader phenomenom that can potentially impact any of the aproximately ten hallmarks of cancer[56]. In ovarian cancer, heterogeneity beyond the genetic component can impact tumor cell subpopulations on cancer hallmarks such as sustained proliferative signaling, activation of the angiogenic switch, genomic instability, and evading immune destruction. In an effort to address this issues, several trials focused on specific tyrosine kinase inhibitors with some of them demonstrating activity against VEGFR [57]. Antiangiogenic drugs have been studied extensively as an addition to the chemotherapy backbone [58], but a clear benefit was seen only in a high risk patient population [59], however novel combinations are under way in order to augment their therapeutic potential in combination with immunotherapy [60] or PARPi [61]. In addition, the combination of PARPi with immunotherapy could be synergistic and is under evaluations in recent clinical trials [62]. Hence, future prospects should incorporate all aspects of cancer heterogeneity together with host and tumor microenvironment related factors.

Evolutionary computational methods in addition to the biomedical, genetic and clinical evidence we had so far can generate evidence based treatment strategies that can be further validated. A framework of tumor dynamics in ovarian cancer predicted the superiority of primary debulking surgery in a low volume disease setting [63], while other analyses focused on optimizing the sequence of chemotherapy in relation to immunotherapy [64] or targeting VEGF-mediated angiogenesis [65], approaches that can help us better understand the development of treatment resistance and design more efficient clinical trials. Characterization of growth and dissemination kinetics could also influence treatment strategies [66], while individual patient quantification of the clonal expansion index provides prognostic information that could further influence treatment intensity [49].

Methods such as high throughput single cell sequencing have recently offered the chance to study intratumor heterogeneity from the perspective of rare subclones [67], and together with novel evolutionary computational methods [68] they offer us the tools to have a real and acurate understanding of disease progression and optimal treatment strategies.

## Data Availability

The data analysed during the current study are available from the corresponding author on reasonable request.

## Abbreviations

CGH: Comparative genomic hybridization
HGSOC: High grade serous ovarian cancer
HR: Homologous recombination
IDS: Interval debulking surgery
LOH: Loss of heterozygosity
NACT: Neoadjuvant chemotherapy
NGS: Next generation sequencing
PARPi: PARP inhibitors
PDS: Primary debulking surgery
SNP: Single nucleotide polymorphism
STIC: Serous tubal intraepithelial carcinomas
TILs: Tumor infiltrating lymphocytes
